# Motion-Aware Interplay between WiGig and WiFi for Wireless Virtual Reality

**DOI:** 10.3390/s20236782

**Published:** 2020-11-27

**Authors:** Sanghyun Kim, Ji-Hoon Yun

**Affiliations:** Department of Electrical and Information Engineering, Seoul National University of Science and Technology, Seoul 01811, Korea; shk0787@seoultech.ac.kr

**Keywords:** wireless virtual reality, WiGig, WiFi, multi-radio, VR

## Abstract

Wireless virtual reality (VR) is a promising direction for future VR systems that offloads heavy computation to a remote processing entity and wirelessly receives high-quality streams. WiGig and WiFi are representative solutions to implement wireless VR; however, they differ in communication bandwidth and reliability. Our testbed experiments show that the performance of WiGig and VR traffic generation strongly correlates with and consequently can be predicted from a user’s motion. Based on this observation, we develop a wireless VR system that exploits the benefits of both links by switching between them and controlling the VR frame encoding for latency regulation and image quality enhancement. The proposed system predicts the performance of the links and selects the one with a higher capacity in an opportunistic manner. It adjusts the encoding rate of the host based on the motion-aware prediction of the frame size and estimated latency of the selected link. By evaluating the testbed data, we demonstrate that the proposed system outperforms a WiGig-only system with a fixed encoding rate in terms of latency regulation and image quality.

## 1. Introduction

Virtual reality (VR) services let a user immerse in a virtual world by enabling the user to explore the virtual space, which is rendered as stereoscopic images, in the way he/she does in the real world, i.e., through head movements [1]. This process is achieved by a VR-dedicated headset device equipped with a head-mounted display (HMD) and a built-in inertial measurement unit (IMU) to capture the user’s head motion. The user sees a stereoscopic image of the virtual world that corresponds to his/her current viewport (estimated from the latest head tracking data) on a display panel at an ultrashort viewing distance (several centimeters) through binocular magnifying lenses to enable a large field of view (FOV).

VR headset systems are generally classified into two types: tethered and untethered. Tethered headsets (e.g., Oculus Rift and HTC VIVE) use a powerful PC (or a gaming console) to process the VR content computations, which offer the highest-quality VR experiences of all types with high resolution and frame rate. However, today’s tethered headsets use cables to transmit display and motion data, and the length, weight, and tension of the required wire harness disturb the user’s mobility, which decreases the immersiveness, restricts the scale of supported services, and creates a tripping hazard. Untethered headsets (e.g., Samsung Gear VR and Oculus Go/Quest) have a processing unit inside the headset (either an attached smartphone or an embedded processor) and provide VR services with no wires; thus, they are portable and convenient but have a lower content quality due to the limited processing power.

A promising direction of evolution for both types of VR headset systems is to offload VR processing to a high-end host (either a local PC as in the tethered case or a cloud or mobile edge) and wirelessly stream the rendered VR image frames to a VR headset, which we call *wireless VR* in this paper. The data traffic of wireless VR has a bidirectional nature; the VR image frames rendered by the host are transferred to the VR headset in the downlink, and the motion (IMU) data of the headset are fed back to the host in the uplink, so that the next VR image frame is rendered for the user’s latest viewport. Due to the ultrashort viewing distance through the magnifying lenses of VR headsets, a high pixel resolution of the VR frames is required (e.g., 4/8K). Moreover, to increase immersion with reduced juddering and motion sickness, a high frame rate (e.g., 90 Hz) is desired. Therefore, delivering a VR video service to VR headsets over wireless connectivity requires a large bandwidth.

WiGig [2] and WiFi [3] are the representative wireless technologies to realize wireless VR. Due to the ultrawide bandwidth (2.16 GHz per channel) of the 60-GHz band and the resulting multi-Gbps transmission speed, WiGig is suitable for streaming high-resolution VR image frames with light compression and has been adopted in the wireless adapter for HTC VIVE headsets [4]. However, the inherent characteristics of the 60-GHz spectrum result in a short transmission distance and unstable connection due to blockage [5], which remain serious issues despite the use of beamforming. High battery consumption is another problem, which results from the use of a higher emission power. The US Federal Communications Commission (FCC) has specified a total maximum transmit power of 500 mW for an emission bandwidth greater than 100 MHz in 54–66 GHz [6] than that used for other unlicensed bands. WiFi has not been considered a viable solution for wireless VR due to the insufficient transmission speed, which inevitably results in *latency* and *motion sickness* [7]. Recently, notable solutions such as onAirVR [8] have shown the feasibility of wireless VR using WiFi. The key to the success of this solution is the adoption of the *Timewarp* technique [9,10], where every received image is reprojected before scan-out according to the latest pose of the user; thus, the inconsistency that the user perceives is minimized. However, WiFi greatly requires compressing VR images to fit them into its limited bandwidth, which results in reduced image quality.

In this paper, we propose a wireless VR solution to exploit the benefits of both WiGig and WiFi in a user-motion- and performance-aware manner for high-quality and reliable VR services, which is called the **M***otion-aware* **W***iGig-***W***iFi* **I***nterplay system for* **V***irtual* **R***eality* (MW${}^{2}$IVR), on the hardware platform currently available in the market, i.e, with off-the-shelf WiGig and WiFi interface modules. The solution is designed to satisfy the following key requirements:

- *High image quality*: The quality of the VR images shown to a user is the first design factor to consider in the cooperative use of both modules, so that the user is immersed in the VR services. Increasing the quality of the encoded images (frames) requires an increased data size. Therefore, the VR system must be designed to be able to transfer large frame sizes as frequently as possible.
- *Regulated latency*: Since the total latency becomes excessive, Timewarp produces noticeable black borders, which disturb the immersion. *Overfilling* [11] is the solution to this problem; it renders an expanded area at the expense of an increased computational load. However, the fluctuating latency results in nonoptimal overfilling, since a specific overfilling factor cannot simultaneously minimize both black borders and computational load under fluctuating latency. Therefore, the cooperative use of both modules should be designed to achieve a given target latency.

MW${}^{2}$IVR jointly manages the dynamic switching between WiGig and WiFi interfaces and the adjustment of the VR content data rate to satisfy the above requirements. To achieve this goal, MW${}^{2}$IVR should find answers to two essential questions: (1) How can the WiGig and WiFi link performance be predicted? (2) How can an appropriate frame size be estimated?

Through testbed experiments, we observe that the WiGig performance greatly depends on the distance and angular direction, while the WiFi performance is stably maintained in a room-scale VR environment. The generated frame size is also proportional to the head angular speed due to the changing user viewport. Based on these observations, MW${}^{2}$IVR is designed to employ the link selection and encoding rate adjustment algorithms to answer the above questions. For link selection, MW${}^{2}$IVR predicts the performance of both links. In particular, the WiGig performance is predicted based on the current distance and head angular direction with respect to the access point. Then, the link with higher capacity is selected in an opportunistic manner. To satisfy the target transmission latency requirement while achieving high image quality, MW${}^{2}$IVR predicts the upcoming frame size based on the relationship with the head speed, estimates the latency for the selected link, and adjusts the encoding rate of the host to bring the latency close to the target.

In summary, the main contributions of our work are listed as follows:

- Design of the prediction scheme for the WiGig throughput and the VR frame size to be generated based on the motion awareness of a VR user,
- Design of the joint control mechanism of interface switching and encoding rate adjustment for enhanced VR frame quality and latency regulation,
- Experimental evaluation of the integrated wireless VR system to show the performance gain of the proposed design over various conventional approaches.

For evaluation, we build a wireless VR system testbed and collect the data of the user motion, link signal levels, and generated frame size. The evaluation of these testbed data shows that MW${}^{2}$IVR outperforms the WiGig-only system with a fixed encoding rate in terms of latency regulation and image quality.

The remainder of the paper is organized as follows: In Section 2, we review the related works. In Section 3, we describe the system model under consideration. Section 4 presents experimental observations of the user motion, wireless performance, and VR traffic generation. The MW${}^{2}$IVR system is described in detail in Section 5, and the experimental evaluation is shown in Section 6. Finally, Section 7 concludes the paper.

## 2. Related Work

There have been recent attempts to design communication and networking schemes for wireless VR in unlicensed spectra. Abari et al. [12] proposed MoVR to solve the signal blocking problem in the 60-GHz band by reflecting signals toward the user. Kim et al. [13] proposed a dynamic/adaptive algorithm that could control the power allocation in 60-GHz transceivers to achieve the time-average energy efficiency for VR data delivery while preserving the queue stabilization. In [14], the feasibility of wireless VR using WiGig was examined through performance measurements and simulation studies. In [15], the feasibility of wireless VR over WiFi was examined via testbed experiments, and the challenges were discussed. Ahn et al. [16] proposed securing timely transmission opportunities by using trigger-based transmission. Tan et al. [17] proposed several enhancement schemes for the WiFi medium access control (MAC) protocol to better support the motion feedback of wireless VR, including prioritizing aged motion data, obtaining motion feedback using reverse direction, and limiting the aggregation size.

There is increasing research on delivering VR services in cellular networks such as 5G systems. Elbamby et al. [18] discussed the challenges and enablers for ultrareliable and low-latency wireless VR, including edge computing and proactive caching in millimeter wave (mmWave) cellular networks. Chen et al. [19] solved a resource management problem in cellular networks for wireless VR, which exploited the potential spatial data correlations among users due to their engagement in the same VR environment to reduce the traffic load in both uplink and downlink. The problem was solved using a machine learning algorithm, which used echo state networks with transfer learning. Guong et al. [20] solved a similar problem using distributed learning in mmWave-enabled wireless networks with mobile edge computing. Dang and Peng [21] solved a joint radio communication, caching and computing decision problems to maximize the average tolerant delay at both mobile VR devices and fog access points. Huang and Zhang [22] proposed a multiuser MAC scheduling scheme with a low-complexity downlink user selection algorithm for VR service in a 5G system; the proposed method includes video frame differentiation and delay-based weight calculation, spatial-frequency user selection, and link adaptation with a dynamic block-error-rate target.

Other research directions for wireless VR include VR content compression methods [23,24], wireless streaming of 360-degree VR video [25,26], and position tracking using WiFi [27]. Li et al. [28] proposed a quality of experience (QoE) model to stream 360-degree VR video in wireless networks. Existing research on the exploitation of multiple radios is also relevant, but most of it has focused on general data communication. Sur et al. [29] proposed a WiFi-assisted 60-GHz link adaptation algorithm that predicted the beam and physical layer (PHY) rate setting and a blockage detection and switching algorithm. E-MICE [30] exploits multiple WiFi radio interfaces in an energy-efficient manner, which activates and deactivates a radio for effective capacity enhancement through machine learning-based prediction algorithms.

## 3. System Model

**Figure 1:**
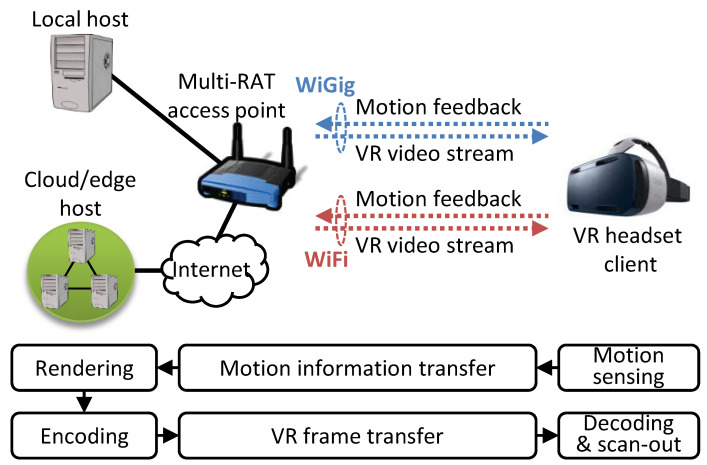
Multi-RAT wireless VR system.

The wireless VR system under consideration is illustrated in Figure 1. A VR headset client is connected to a remote processing host, which can be a local PC or a cloud/mobile edge, via a multiradio access technology (RAT) access point (AP) that supports WiGig and WiFi. We assume that the headset client uses one link (WiGig or WiFi) at a time, not both simultaneously, due to excessive energy consumption. The processing chain and data flow of wireless VR are also illustrated in Figure 1. To track a user’s viewport in real time, the VR headset continuously measures the user’s head pose (yaw, pitch, and roll orientations) with its built-in IMU and reports it to the host through uplink transmission. The host generates VR image frames based on the reported motion data and streams them to the headset client through downlink transmission. Due to the insufficient bandwidth of the network or link, the VR image frames are encoded as a video stream using a compression codec (e.g., H.265). We call each compressed VR frame a VR video frame. The headset client decodes the received frames and scans them out on the display panel. A timewarp may be applied to them before they are scanned out. Each of these processing blocks introduces some latency. In particular, the latency components of motion report transfer, encoding, frame transfer, and decoding are newly introduced in wireless VR.

In our testbed, a local PC is used as a processing host. A Netgear Nighthawk X10 and an Acer Travelmate laptop (equipped with the Sparrow 11ad module) are used. An HTC VIVE headset is connected to the laptop via HDMI and USB cables. The motion data of the headset are captured and transferred to the host using VirtualHere software [31]. The compression codec is H.265 from the *x265* library [32], and the streaming protocol is the real-time transport protocol (RTP) over UDP.

## 4. Experimental Observations on the Impact of User Motion

### 4.1. Impact of User Motion on the Wireless Performance

To observe the performance of WiGig and WiFi in wireless VR scenarios, we perform throughput measurement tests. Within a VR service, the VR user walks around and rotates his/her head to explore the virtual world. In other words, when the user moves, the position and directional angle of the headset with respect to the connected AP change. For performance measurement, we establish a line-of-sight WiGig/WiFi connection between the AP and the client, run iPerf tests, and observe throughput changes for varying distances and directional angles.

**Figure 2:**
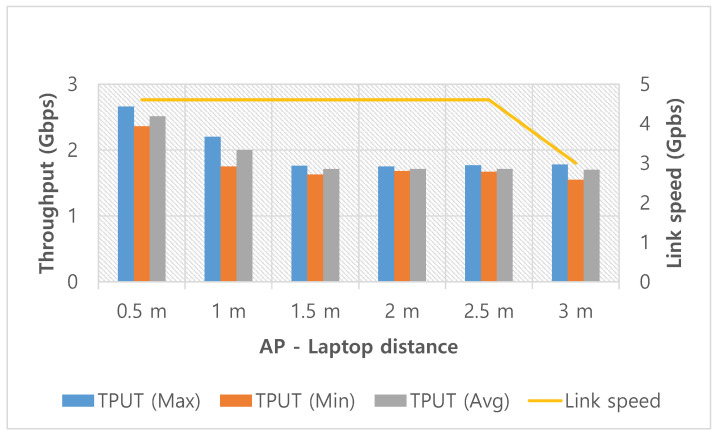
Throughput performance of WiGig at various distances.

The throughput performance of WiGig for varying distances is shown in Figure 2. In this experiment, the lid of the laptop where antennas are embedded faces the AP, which corresponds to the front direction or zero-degree angle. When the distance is 0.5 m, the throughput is measured as 2.5 Gbps. However, at 1 m, the throughput significantly decreases to 2 Gbps. Beyond 1 m, the throughput is approximately 1.5 Gbps. When the distance increases from 1.5 m to 3 m, no meaningful change in the throughput is observed. These experimental results imply that WiGig’s performance is sensitive to the distance within a nearby area but becomes less sensitive once the distance exceeds a certain threshold. Over 3 m, we experienced unpredictable/irregular disconnections of the WiGig interface. If the WiGig connection is made stable, it will be meaningful to investigate the system performance in a wider service area.

**Figure 3:**
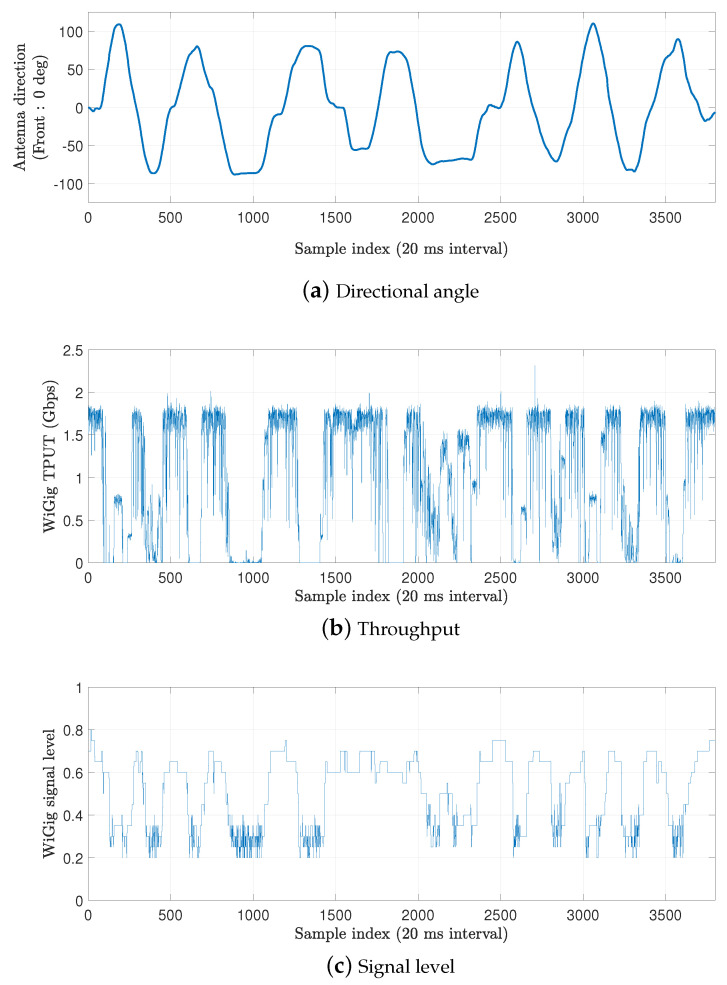
Changes in the directional angle, throughput, and signal level for WiGig with time.

**Figure 4:**
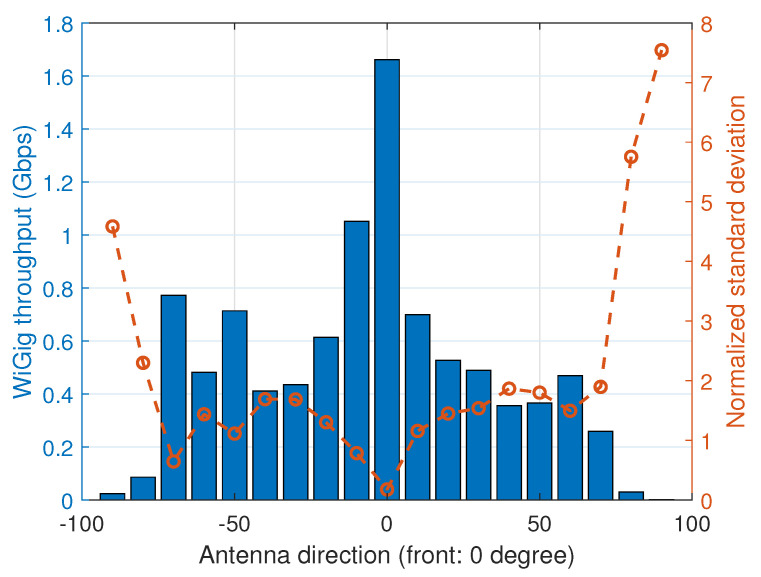
Mean and normalized standard deviation of WiGig throughput for varying antenna directions.

In the second experiment, we continuously change the orientation of the laptop during the *iPerf* [33] test at a distance of 2 m, which is more similar to real wireless VR scenarios. During the experiment, we measure the directional angle of the client and the signal level of the WiGig module (as reported by the wil6210 driver [34]). The collected data and throughput measurement over time are shown in Figure 3; the mean and normalized standard deviation (divided by the mean) of the throughput for varying antenna direction are given in Figure 4. In the figures, we observe a correlation between the directional angle (yaw value of the IMU data) and the performance of WiGig. The throughput is at the highest point when the direction is toward the front (zero degree). However, when the client rotates, the throughput dramatically decreases and, moreover, the normalized standard deviation increases (implying higher relative fluctuation). The signal level of WiGig also strongly correlates with the direction; it is measured to be highest near zero degree and almost proportionally decreases with the angle increase. We expect that this decrease results from imperfect and slow beamforming operations.

**Figure 5:**
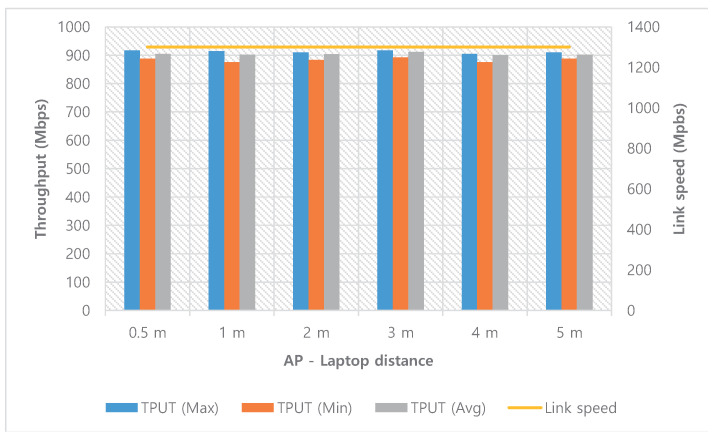
Throughput performance of WiFi at various distances.

Unlike WiGig, the performance of WiFi is not sensitive to user motion at the room scale. In the same environment as the WiGig test (performed at midnight to minimize interference), the throughput performance of WiFi remains unchanged with distance and direction, as shown in Figure 5. Although the signal strength of WiFi decreases with increasing distance, the link speed (PHY rate) of WiFi does not change. However, the throughput of WiFi is only one-third the maximum throughput that WiGig can achieve.

### 4.2. Impact of User Motion on VR Traffic

Due to the limited bandwidth of wireless connectivity, the wireless VR system under consideration transfers a stream of encoded VR frames to a user headset. The principle of video encoding is to extract the differential information between frames as motion vectors; larger differences between frames result in more information, which increases the size of the resulting encoded frame. If we can predict the sizes of upcoming frames, this will be important information in MW${}^{2}$IVR for adaptive operation.

**Figure 6:**
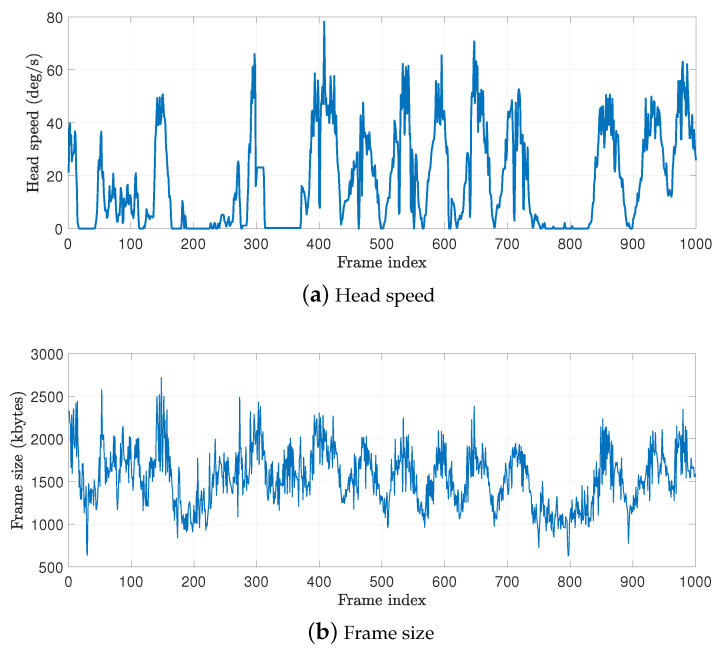
Changes in the head angular speed and encoded frame size with time.

In wireless VR, the major source of differences between frames is the user’s motion. To investigate the relationship between the user motion and the amount of generated VR traffic to be delivered, we simultaneously collect both data during the playing of VR content in our wireless VR system. Figure 6 shows the head angular speed and frame size with changing frame indices. We observe an apparent correlation between the two quantities. The peaks of the head speed match those of the frame size. The stationary periods of the user (when the head speed is close to zero) are also synchronized with the valleys of the frame size.

**Figure 7:**
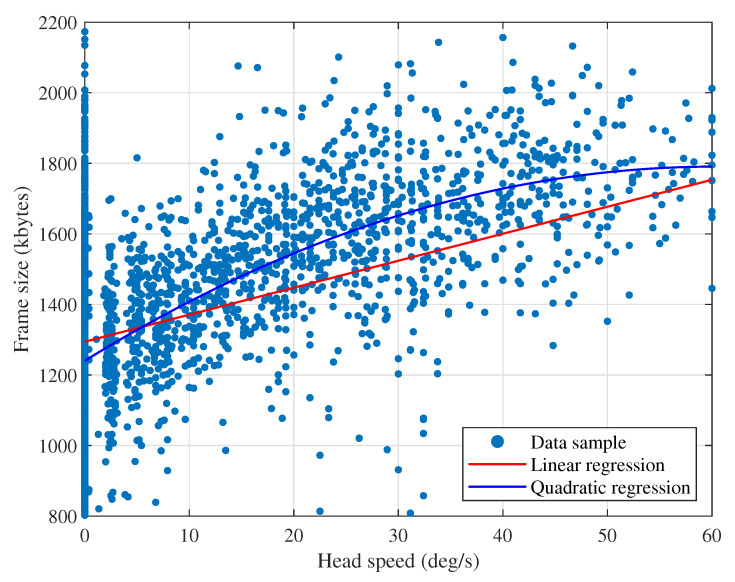
Scatter plot of the frame size vs. head angular speed.

To quantitatively observe the correlation between the two parameters, we show the scatter plot in Figure 7; The red line and blue curve are the linear and quadratic regression results, respectively, of the data samples showing a trend. The frame size is clearly proportional to the head speed. In statistics, the *p*-value is commonly used as a measure of correlation; it is defined as the probability of obtaining the observed correlation under the hypothesis of no correlation, i.e., the true correlation is zero. Then, a sufficiently small *p*-value (typically less than 0.05 or 0.01) implies that such a correlation is unlikely observed under this hypothesis, and the new hypothesis of a significant correlation should be accepted as true. For the samples in the figure, the *p*-value is $2.7\times 10^{-204}$; thus, a strong correlation between two variables is concluded.

The VR content may have moving objects in its scenes. Therefore, although the user is in a steady state, the frame size may change with time. The user’s motion will cause additional changes in the frame size. The amount of VR traffic to transmit will not increase immediately after the user’s motion due to the latency components between the occurrence of the user’s motion and the transmission of the corresponding VR traffic, such as motion data transfer, content simulation, rendering, and encoding.

## 5. MW${}^{2}$IVR

**Figure 8:**
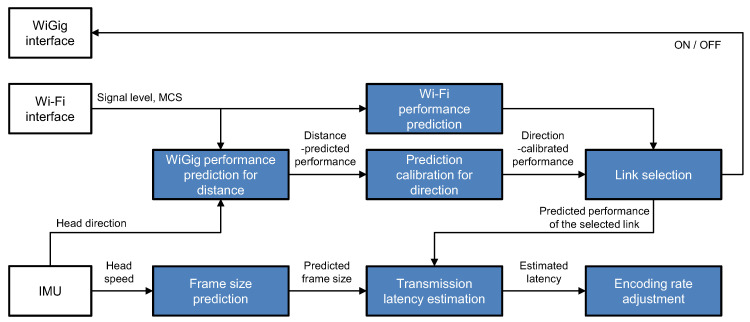
Building blocks of MW${}^{2}$IVR.

Based on the observations in the previous sections, we know that WiGig and WiFi are complementary to each other, so cooperatively utilizing them can help to achieve high image quality and regulated latency. MW${}^{2}$IVR is designed to achieve this goal using two algorithms: (1) link selection (between WiGig and WiFi) for higher throughput and (2) encoding rate adjustment to satisfy the target latency for the selected link. The building blocks of MW${}^{2}$IVR are illustrated in Figure 8.

We will describe each algorithm of MW${}^{2}$IVR in detail.

### 5.1. Link Selection

MW${}^{2}$IVR selects the link with the highest predicted performance between WiGig and WiFi. Let $R_{\mathrm{WiGig}}$ and $R_{\mathrm{WiFi}}$ be the predicted throughput performance of the WiGig and WiFi links, respectively. The resulting throughput of the wireless connectivity, which we denote by $R^{*}$, is obtained as

(1) $$R^{*}=\max\left\{R_{\mathrm{WiGig}},R_{\mathrm{WiFi}}\right\}.$$

When the WiFi link is in use for VR streaming, MW${}^{2}$IVR switches to the WiGig link if the predicted WiGig performance is better than the WiFi performance. Likewise, while the WiGig link is in use, MW${}^{2}$IVR switches back to the WiFi link if the WiFi performance becomes better than the WiGig performance.

According to the experimental observations in Section 4.1, the WiFi performance remains stable in a room-scale environment. Therefore, MW${}^{2}$IVR considers the performance of the WiFi to be constant. MW${}^{2}$IVR uses the WiFi link as a default link for stable service and does not turn it off due to its relatively low power consumption. Thus, the WiFi performance is predicted from its modulation and coding scheme (MCS) in use.

When the WiGig link is not in use for VR streaming, it is turned off to reduce the power consumption of the client. The average power consumption of the WiGig interface was measured using the Wattman power consumption analyzer (HPM-100A) [35] as 8.4 watts higher than the idle state (both WiGig and WiFi are turned off), 4.3 watts higher than WiFi at 2.4 GHz, and three watts higher than WiFi at 5 GHz. The average off-to-on delay of the WiGig interface was around 300 ms (the average of ten trials). MW${}^{2}$IVR switches from WiFi to WiGig after the WiGig interface is confirmed connected. Therefore, it is necessary to estimate the performance of the WiGig link when it is deactivated. For this purpose, MW${}^{2}$IVR uses a two-step approach as follows. In the first step, it estimates the maximum WiGig performance for a given distance assuming that the client faces toward the front. The current distance is estimated based on the WiFi signal strength or more accurately obtained by the positional information if the headset is equipped with positional tracking. In the next step, MW${}^{2}$IVR applies a scaling factor to the maximum performance to consider the direction effect. Finally, MW${}^{2}$IVR obtains the predicted throughput performance of the WiGig link at the given distance and direction. The determination of the information necessary for prediction is based on the a priori determination of the WiGig performance.

### 5.2. Encoding Rate Adjustment

MW${}^{2}$IVR adjusts the data rate of encoding on the host side such that the target transmission latency is satisfied for the selected link. Let *L* be the mean size of the generated VR frames. Latency *D* in delivering a VR frame to the client using the selected link at rate $R^{*}$ is

(2) $$D=\frac{L}{R^{*}}.$$

MW${}^{2}$IVR aims to constrain the actual latency to be near the target latency $D_{t}$.

**Figure 9:**
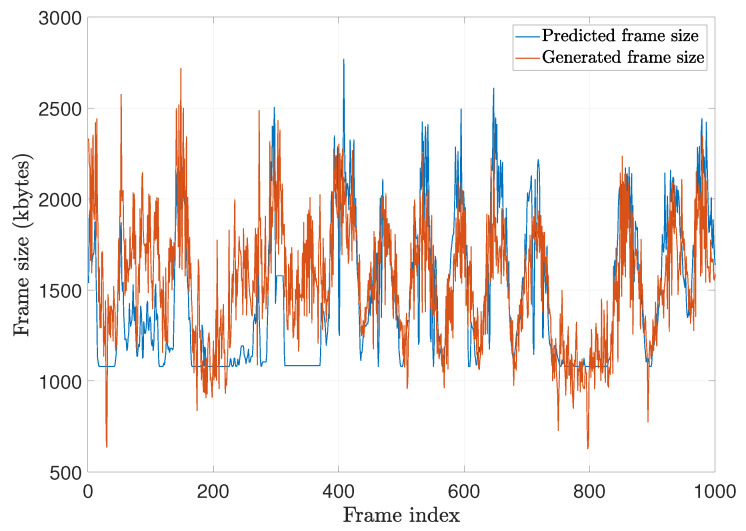
Predicted frame size vs. generated frame size.

As observed in Section 4.2, the frame size is affected by the head motion; thus, MW${}^{2}$IVR must predict the frame size to be generated and adjust it accordingly. MW${}^{2}$IVR predicts the frame size using the head angular speed obtained from the headset’s IMU data. The VR content has a base size $L_{base}$ even when the head speed is zero. When the head speed increases, the frame size also increases. Based on this relationship, MW${}^{2}$IVR predicts the upcoming frame size as

(3) $$L_{predicted}=\alpha \cdot v+L_{base},$$

where *v* is the head speed and α is the scaling constant, which is found as the increase rate of the frame size for changing *v*, and α and $L_{base}$ can be predetermined or continuously calibrated during VR service. The accuracy of the frame size prediction is illustrated in Figure 9. α is set to 180 bytes/(deg/s), and $L_{base}$ is 1080 kbytes. The figure shows that the predicted frame size matches the generated frame size overall. Relatively large errors are observed when the user is in a steady state because the VR objects still move, which affects the frame size. We denote the expected latency for the predicted frame size $L_{predicted}$ as $D_{expected}$.

To satisfy the target latency $D_{t}$ for the predicted frame size, MW${}^{2}$IVR adjusts the encoding rate of the host’s encoder. MW${}^{2}$IVR increases the encoding rate if $D_{expected}$ is lower than $D_{t}$ and decreases it otherwise. The encoding configurations of the encoder cannot be changed in the middle of a group of pictures (GOP). Thus, the encoding rate remains unchanged until a new GOP begins. Table 1 illustrates the video quality in terms of the peak signal-to-noise ratio (PSNR) for varying target bit rates when the encoder ×265 is used. As the target bit rate is decreased, the PSNR is also decreased. Another parameter of ×265 to control the generated frame size is the quality, as illustrated in Table 2. In Equation (2), when the encoder generates smaller frames, the latency is decreased. However, from the results in the tables, a smaller frame size implies a lower VR video quality. Therefore, MW${}^{2}$IVR adjusts the encoding rate to better satisfy the target latency instead of minimizing the latency.

## 6. Performance Evaluation

In this section, we evaluate the performance of MW${}^{2}$IVR in comparison with various systems, using data from our wireless VR system testbed. The performance of MW${}^{2}$IVR is evaluated in terms of the link latency (Equation (2)) and delivered frame size.

### 6.1. Evaluation Configuration

To evaluate the performance of MW${}^{2}$IVR and other systems in the same environment, including the user motion, we record the IMU data during the playing of the interactive VR content *Great Power* [36] and the throughput and signal levels of both links in the log files. Then, the recorded IMU data and other logged data are fed into the host of MW${}^{2}$IVR and the other systems in comparison. We set the GOP size of the encoder as half of the frame rate, so that MW${}^{2}$IVR can update the encoding rate twice per second. We set the target latency ($D_{t}$) of MW${}^{2}$IVR to 11 ms, which is the average latency of the legacy WiGig-only system. The WiFi signal levels of –30, –35, –39, –42, –45, and –47 dBm are interpreted as distances of 0.5, 1, 2, 3, 4, and 5 m and mapped to the throughput scaling factors for the distances of 1, 0.74, 0.71, 0.66, 0.64, and 0.64, respectively. The throughput scaling factors for the head directional angles of zero, <25, <45, and ≥45 degrees are 1, 0.78, 0.5, and 0. These parameter configurations are based on the experimental results obtained in the testbed. α and $L_{base}$ for the frame size prediction in Equation (3) are set to be identical to those in Figure 9: 180 bytes/(deg/s) and 1080 kbytes, respectively. The encoding rate determined by MW${}^{2}$IVR is set to the target bit rate of the x265 encoder. For comparative evaluation, conventional techniques (other systems) are represented into the following classes:

- Legacy VR with a fixed interface: The wireless interface in use is not changed during VR service. The encoding rate is fixed as well. The cases of WiGig-only and WiFi-only are considered.
- Interface switching: Switching between WiGig and WiFi interfaces is made for higher throughput during VR service. The switching algorithm of MW${}^{2}$IVR without encoding rate adjustment is considered.
- Encoding adjustment: The encoding rate of a VR service is adjusted for latency regulation but without interface switching. The adjustment algorithm of MW${}^{2}$IVR is considered, and thus accompanies the proposed motion-aware VR traffic prediction scheme. The cases of WiGig-only and WiFi-only are considered.

### 6.2. Frame Transmission Latency

**Figure 10:**
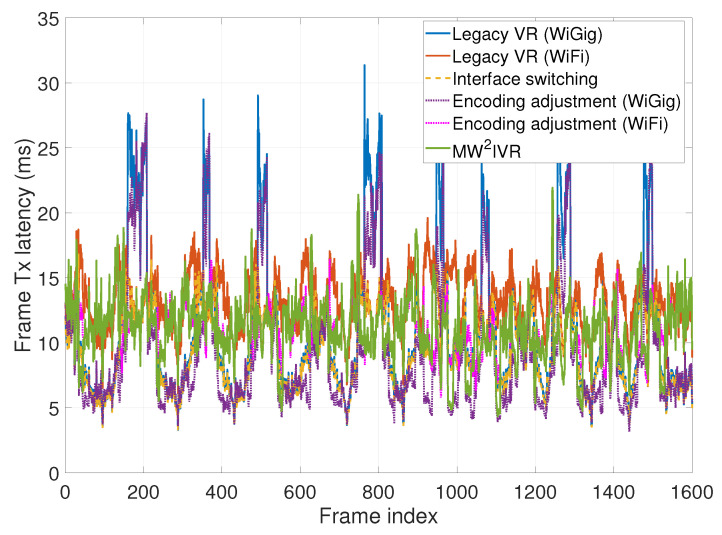
Frame transmission latency over time.

Figure 10 shows the frame transmission latency over time. In the figure, MW${}^{2}$IVR achieves the regulated latency around the target (11 ms), while the other systems except legacy VR (WiFi) show fluctuating latency. The reason is that the head motion simultaneously increases the upcoming frame size and reduces the throughput of WiGig, which amplifies the latency increase. The legacy VR (WiGig) and encoding adjustment (WiGig) systems cannot handle such a latency increase, while MW${}^{2}$IVR successfully handles it by an opportunistic switch to WiFi and an encoding rate adjustment, thus having the latency upper-bounded by 22 ms and mostly under 15 ms. The legacy VR (WiGig) and interface switching systems often have lower latency than MW${}^{2}$IVR, which, however, do not increase the enhanced image quality due to the fixed encoding rate. The legacy VR (WiFi) and encoding adjustment (WiFi) systems achieve regulated latency due to the stable throughput performance of the WiFi interface in the entire service area.

**Figure 11:**
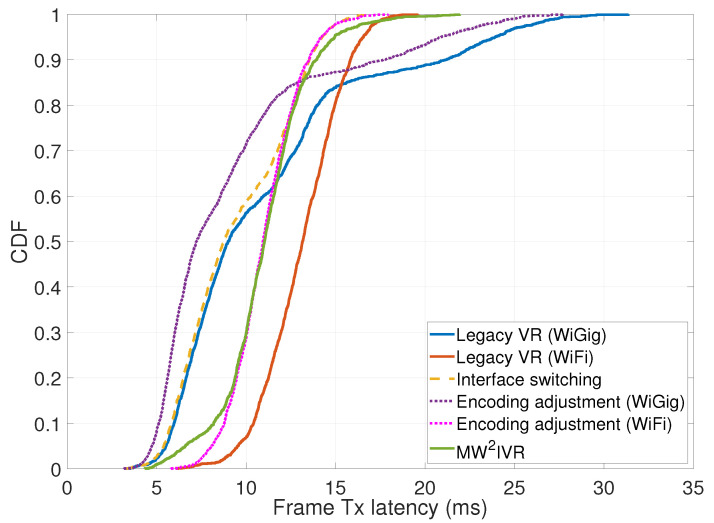
Cumulative distribution function of the frame transmission latency.

The cumulative distribution function (CDF) in Figure 11 supports this observation. The averages for both MW${}^{2}$IVR and legacy VR (WiGig) systems are similarly obtained as 11.3087 ms (MW${}^{2}$IVR) and 11.0802 ms (legacy VR (WiGig)). However, the latency of the legacy VR (WiGig) system spans a wide range, which implies a failure of latency regulation. The latency of MW${}^{2}$IVR is distributed in a narrower range around the target latency. The legacy VR (WiFi) system shows regulated, but higher latency than MW${}^{2}$IVR. The interface switching system shows similar latency to MW${}^{2}$IVR for 25% of the samples, but lower latency for the rest when the link condition of WiGig is good (since the encoding rate is fixed). The encoding adjustment (WiFi) system shows almost the same pattern of latency distribution as MW${}^{2}$IVR.

### 6.3. Generated Frame Size

**Figure 12:**
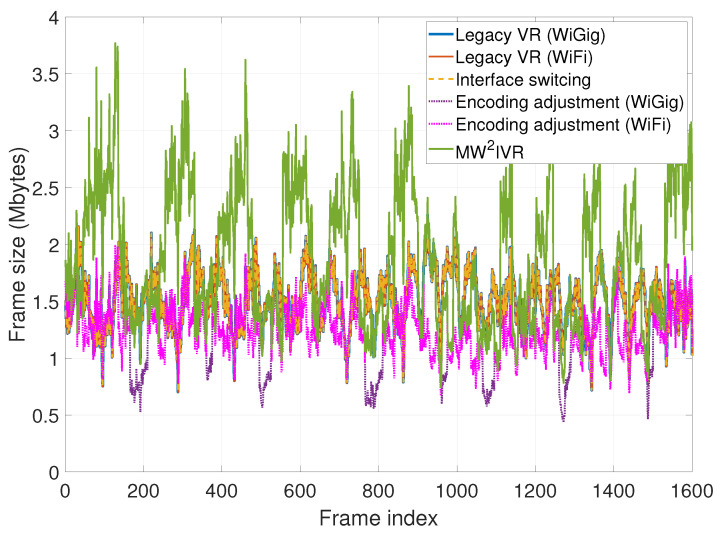
Generated and delivered frame sizes over time.

The generated and delivered frame sizes over time are shown in Figure 12. The systems except encoding adjustment (WiGig) have similar lower bounds of the frame size to each other. However, MW${}^{2}$IVR achieves occasional large frame sizes and consequently a higher image quality due to the increase in encoding rate. This increase in the frame size occurs when the user motion slows, since the throughput of the WiGig link increases; thus, MW${}^{2}$IVR has room to increase the encoding rate. Despite such opportunistic large increases in the frame size, MW${}^{2}$IVR regulates the latency, as shown in the latency results. The figure also shows that the frame size increase for MW${}^{2}$IVR is somewhat synchronized with the frame size and latency decrease of the legacy VR (WiGig) system. Hence, the near-zero head motion makes room for the wireless link to deliver more data, but the other systems do not fully utilize it. The frame sizes of the legacy VR and interface switching systems show the same pattern of increasing and decreasing as they use a fixed encoding rate. The frame size of the encoding adjustment (WiGig) system increases similarly with MW${}^{2}$IVR when the link condition of WiGig is good, but decreases even lower than MW${}^{2}$IVR whenever the the link condition of WiGig gets poor, at the expense of degraded VR frame quality. MW${}^{2}$IVR successfully handles such a poor link condition of WiGig by switching to WiFi, thus limiting quality degradation.

**Figure 13:**
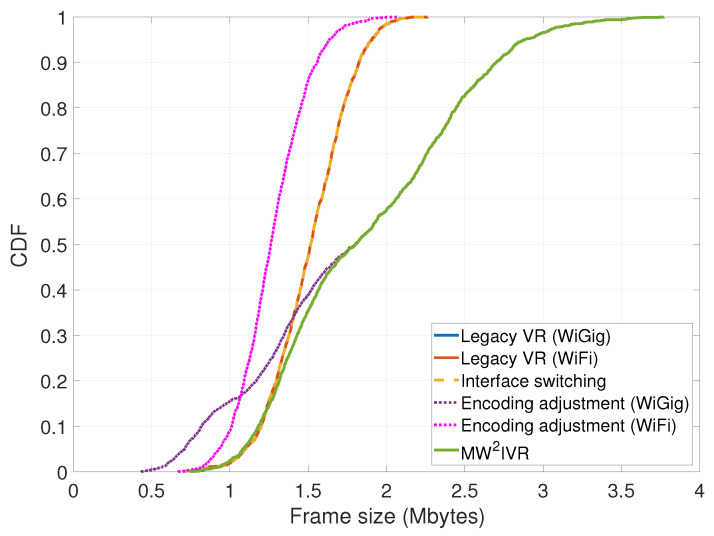
Cumulative distribution function of the generated and delivered frame sizes.

The CDF of the frame size is shown in Figure 13. MW${}^{2}$IVR has larger sizes than the legacy VR and interface switching systems in 80% of the frames, than the encoding adjustment (WiGig) system in 65% of the frames, and than the encoding adjustment (WiFi) system in all frames. In other words, MW${}^{2}$IVR applies a higher encoding rate for a majority of the frames than the other systems. Specifically, MW${}^{2}$IVR achieves a 25% or greater increase in frame size for 40% of the frames than the legacy VR and interface switching systems. The average frame size is 1.883 Mbytes for MW${}^{2}$IVR and 1.509 Mbytes for the legacy VR and interface switching systems (the encoding quality is fixed as 50% of the maximum and 2.2 Mbytes is the maximum frame size of the considered VR content under this setting).

## 7. Conclusions

We developed a wireless VR system that incorporated WiGig and WiFi links for latency regulation and image quality enhancement, which is called MW${}^{2}$IVR. Through testbed experiments, we observed that the WiGig performance greatly depended on the distance and directional angle of the head. We also observed that the generated frame size was affected by the head speed. Based on these observations, MW${}^{2}$IVR was designed to predict the performance of both links and select the WiGig link in an opportunistic manner. MW${}^{2}$IVR adjusts the encoding rate of the host based on the motion-aware prediction of the frame size and the estimated latency for the selected link. By evaluating the testbed data, we demonstrated that MW${}^{2}$IVR outperformed the WiGig-only system with a fixed encoding rate in terms of latency regulation and image quality.

## Figures and Tables

**Table 1:** Target bit rate vs. PSNR.

Target Bit Rate (%)	PSNR	Target Bit Rate (%)	PSNR
100	-		
83	46.06025	27	42.0448
67	45.36735	17	40.1181
50	44.38812	10	39.7944
33	42.9074	3	31.6792

**Table 2:** Encoding quality vs. frame size.

Quality	Frame Size (%)
100	100
80	84.1
50	61.8
30	48.7
10	39.1

## Abbreviations

The following abbreviations are used in this manuscript:

AP	Access Point
CDF	Cumulative Distribution Function
FOV	Field Of View
GOP	Group Of Pictures
HMD	Head-Mounted Display
IMU	Inertial Measurement Unit
MAC	Medium Access Control
MCS	Modulation and Coding Scheme
PHY	Physical Layer
PSNR	Peak Signal-to-Noise Ratio
QoE	Quality of Experience
RAT	Radio Access Technology
RTP	Real-time Transport Protocol
VR	Virtual Reality
